# Supplementary figures and images for: RNA fusion in human retinal development
Source: eLife. 2024 Jan 2;13:e92523. doi: 10.7554/eLife.92523 (PMC10890785; doi:10.7554/eLife.92523)

Day 60 organoids

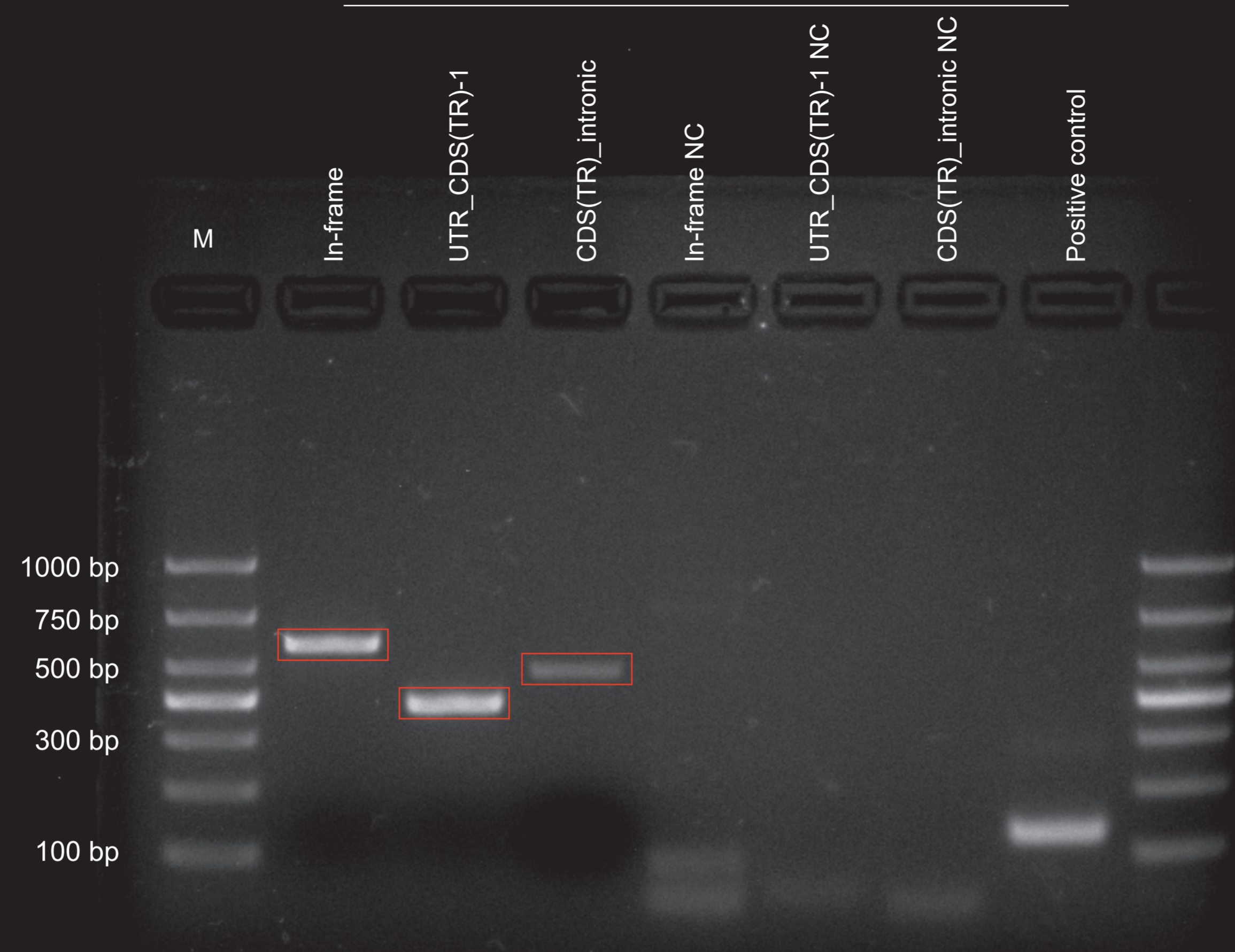

Supplement: Figure 4—source data 2. [file elife-92523-fig4-data2.pdf]

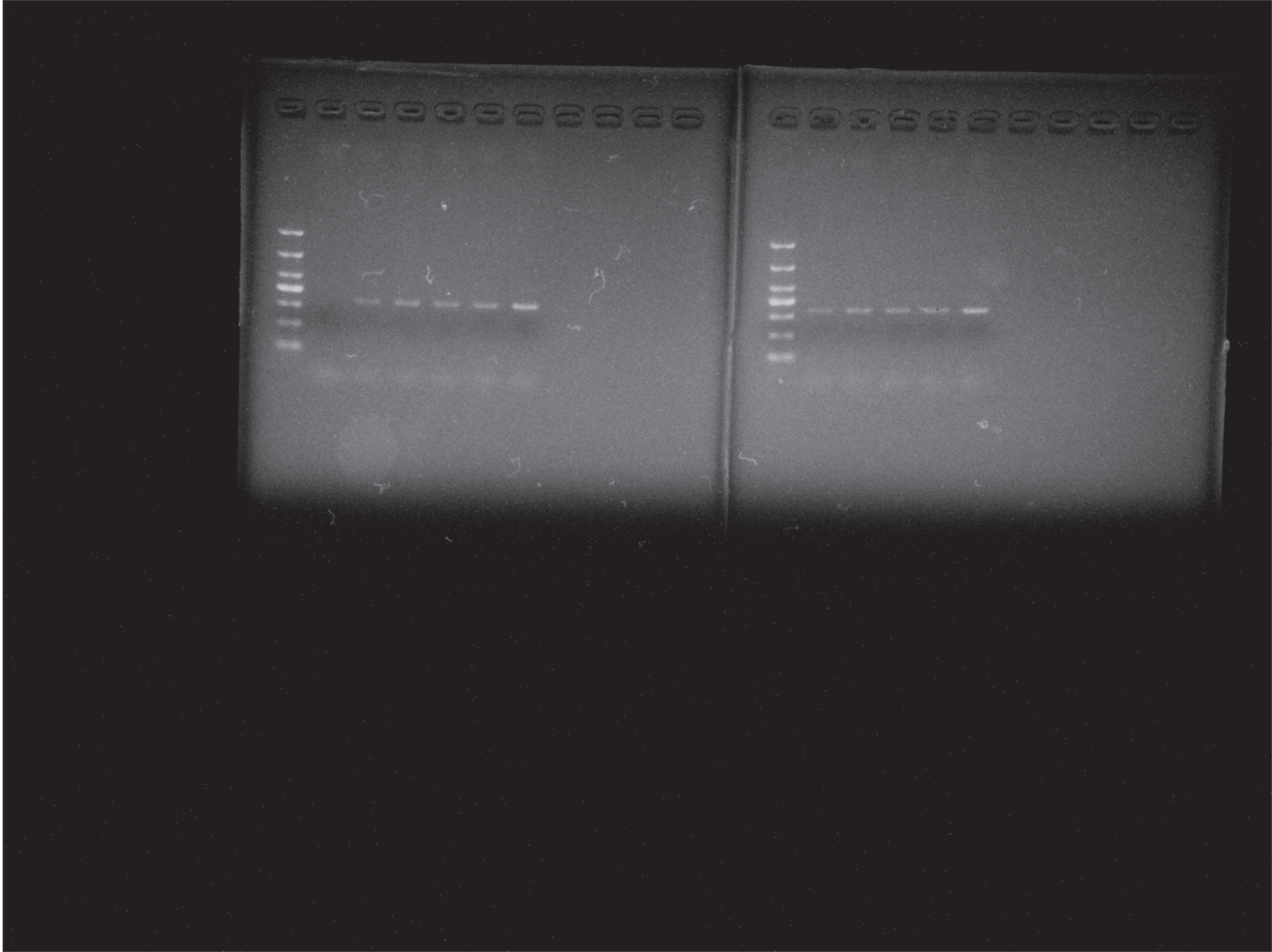

Supplement: Figure 5—source data 1. [file elife-92523-fig5-data1.pdf]

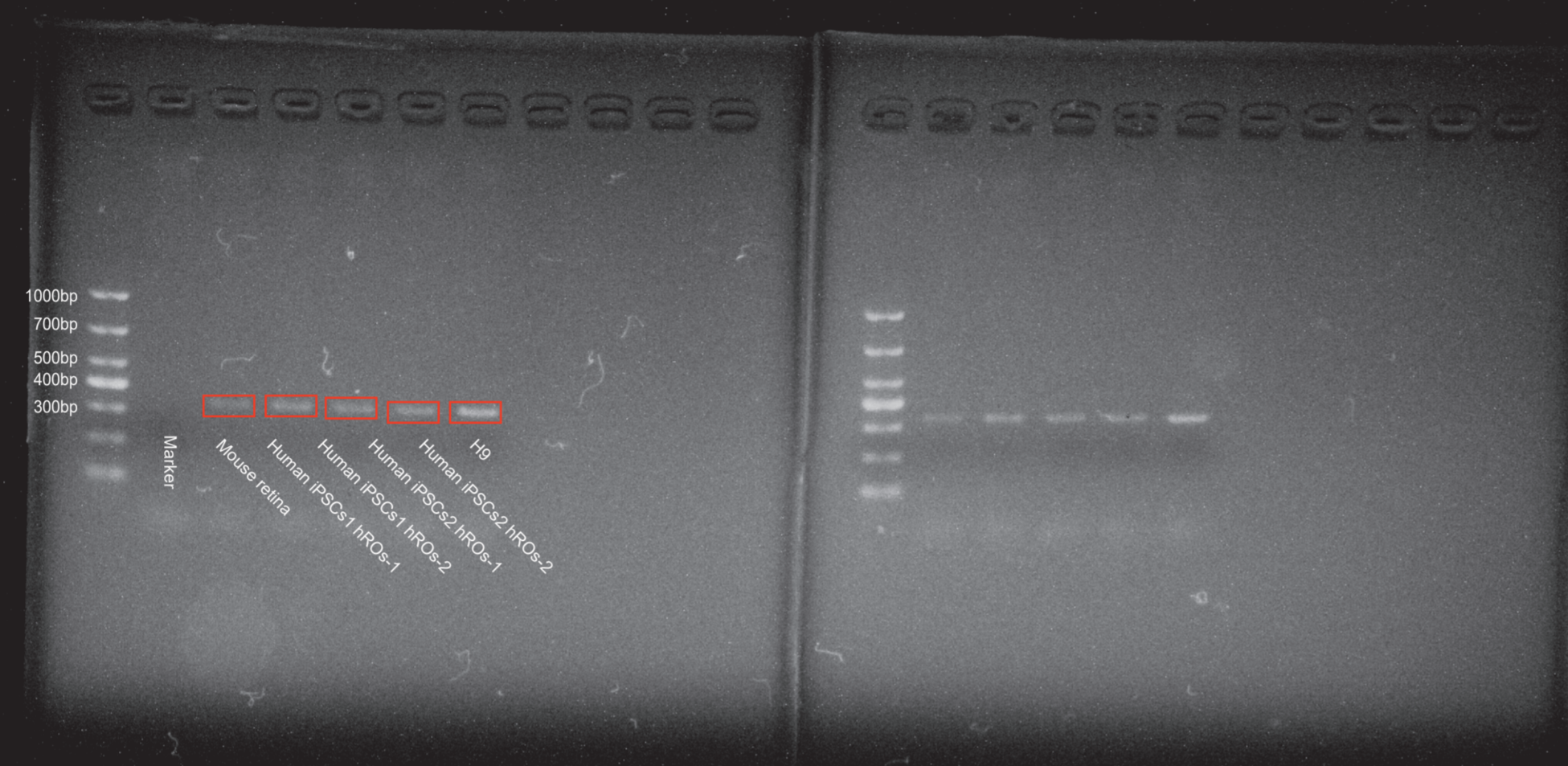

Supplement: Figure 5—source data 2. [file elife-92523-fig5-data2.pdf]

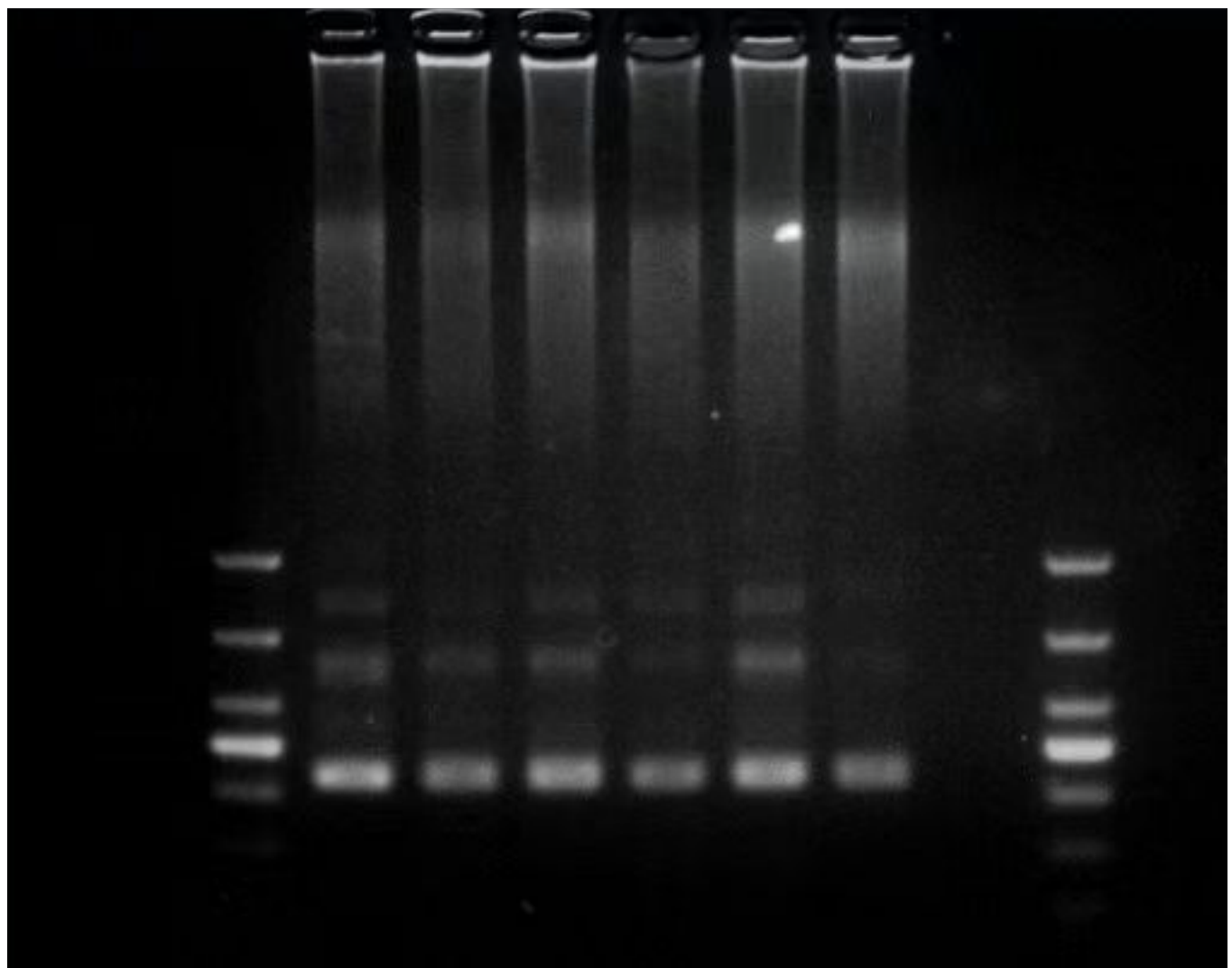

Supplement: Figure 5—source data 3. [file elife-92523-fig5-data3.pdf]

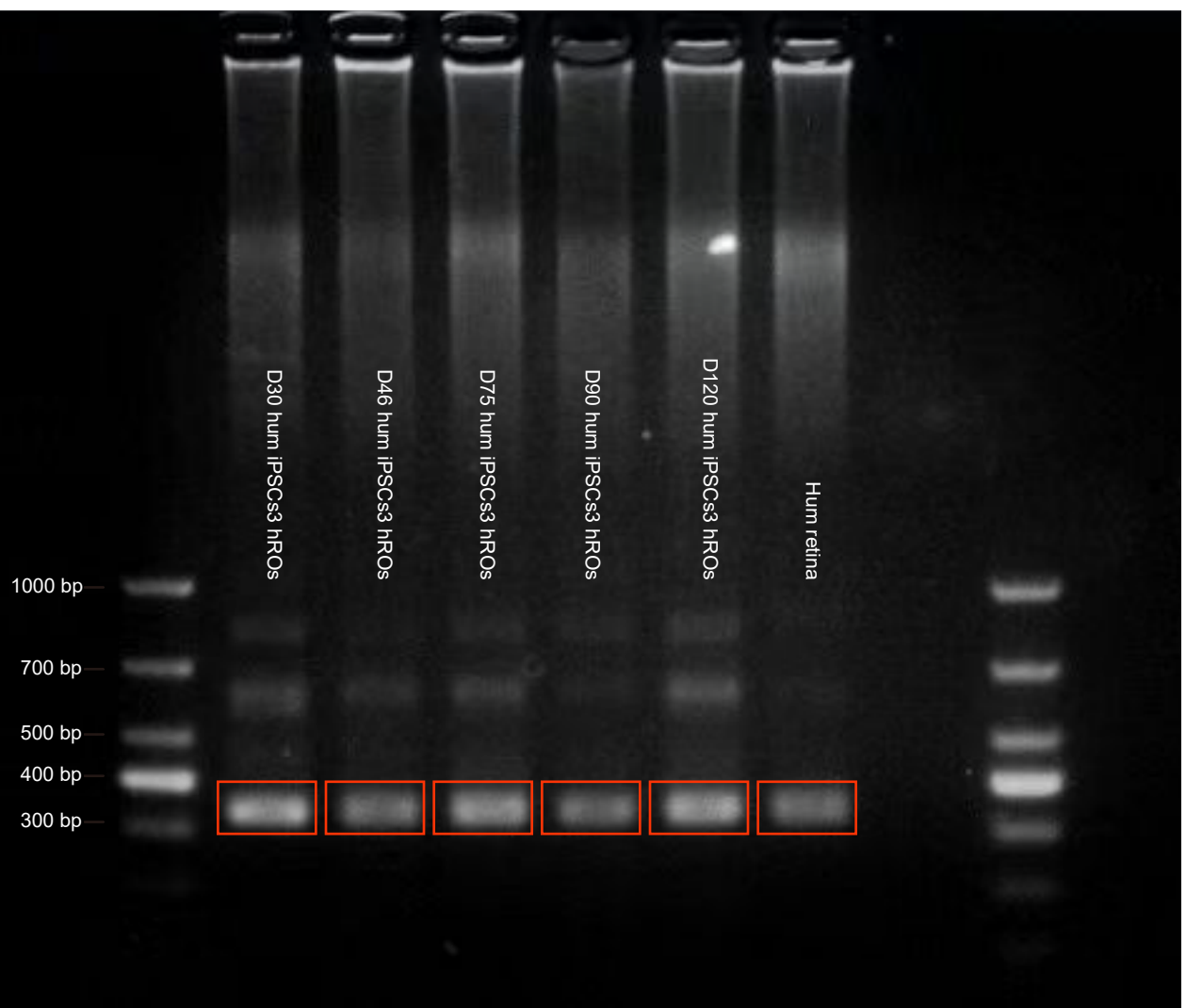

Supplement: Figure 5—source data 4. [file elife-92523-fig5-data4.pdf]
