# Supplementary material for: RNA fusion in human retinal development
Source: eLife. 2024 Jan 2;13:e92523. doi: 10.7554/eLife.92523 (PMC10890785; doi:10.7554/eLife.92523)

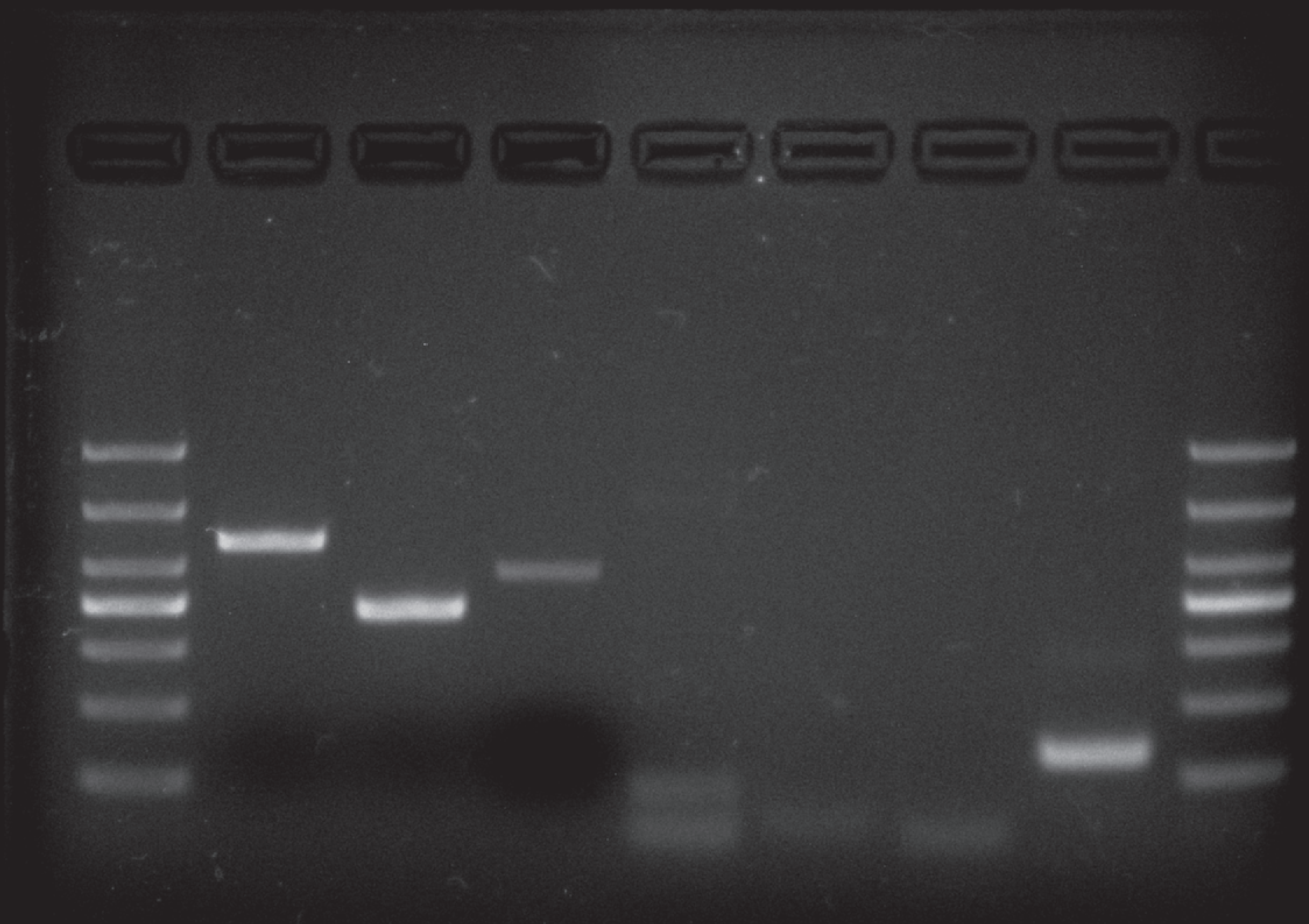

Supplement: Figure 4—source data 1. [file elife-92523-fig4-data1.pdf]
